# Collagen Gene Variants and Anterior Cruciate Ligament Rupture in Italian Athletes: A Preliminary Report

**DOI:** 10.3390/genes14071418

**Published:** 2023-07-09

**Authors:** Myosotis Massidda, Laura Flore, Marco Scorcu, Giovanni Monteleone, Alessandra Tiloca, Massimiliano Salvi, Filippo Tocco, Carla M. Calò

**Affiliations:** 1 Department of Medical Sciences and Public Health, University of Cagliari, 09042 Monserrato, Italy; filippo.tocco@unica.it; 2 Department of Sciences of Life and Environment, University of Cagliari, 09042 Monserrato, Italy; laura.flore@unica.it (L.F.); cmcalo@unica.it (C.M.C.); 3 Italian Federation of Sports Medicine (FIMS), CR Sardegna, 00196 Rome, Italy; mscorcu@tiscali.it; 4 Department of Biomedicine and Prevention, University of Rome Tor Vergata, 00133 Rome, Italy; giovanni.monteleone@uniroma2.it (G.M.); altilok@yahoo.it (A.T.); 5 Korian Kineteca Sardegna, 09045 Quartu Sant’Elena, Italy; massimiliano.salvi@tin.it

**Keywords:** ACL rupture, polymorphisms, COL genes

## Abstract

Several studies have investigated the role of genetics in anterior cruciate ligament (ACL) rupture, often returning conflicting results. The present pilot study aimed to analyze the association between six Single Nucleotide Polymorphisms (SNPs) (rs1800012; rs12722; rs13946; rs240736; rs970547; and rs4870723, located on the *COL1A1*, *COL5A1*, *COL12A1*, and *COL14A1* genes), and ACL rupture, among Italian athletes. A hypothesis-driven association study was conducted. In total, 181 male and female athletes (*n* = 86 injured; *n* = 96 non-injured) were genotyped for the prioritized variants. All polymorphisms were genotyped using PCR RFLP, with the only exception being the rs1800012 on the *COL1A1* gene, which was detected using MTPA PCR. The allele frequency distribution fell within the worldwide range. Despite the evident population variability, no selective pressure signals were recorded using PBS analysis. No significant difference was detected between the cases and controls for any of the SNPs (rs1800012; rs13946; rs240736; rs970547, and rs4870723) included in the analyses (*p* > 0.008, Bonferroni-adjusted for multiple comparisons). Moreover, no significant differences were found when males and females were assessed separately. Further investigations based on a larger sample size are needed, in order to draw solid conclusions for the influence between collagen genes and ACL rupture.

## 1. Introduction

Anterior cruciate ligament rupture (ACL) is one of the most common non-contact musculoskeletal injuries among athletes, especially in high-impact sports [1].

Although many intrinsic and extrinsic factors for ACL rupture have been identified, the exact etiology of this type of injury is still not fully understood [2].

Family predisposition is one of the main genetic risk factors for non-contact musculoskeletal lesions, and the incidence of ACL rupture among first-degree relatives of patients with ACL rupture is more than twice as high as among first-degree relatives of those without injuries [3], suggesting that genetic factors play a role in ACL rupture. Furthermore, new pieces of evidence have been emerging about the potential role of genetic and hormonal profiles, which may substantially alter the structural properties of the ACL, making it more or less vulnerable to failure induced by external loading [4].

The susceptibility of female athletes to cruciate ligament rupture has been studied [5], observing a 3–6 times higher incidence of injury in female athletes than in male athletes [6,7]. These results suggest the influence of female sex hormones on the composition and mechanical properties of the cruciate ligament [8], although anatomical causes cannot be excluded [9,10].

The anterior cruciate ligament is a dense band of connective tissue composed of numerous collagen fibers, such as the collagens encoding the α1 chains of types I, V, and XII that make up most of the solid component of the ligaments [11], and play important roles in normal collagen fibrillogenesis [12,13,14].

Several studies have identified an association between some polymorphisms in the *COL1A1*, *COL5A1*, *COL12A1*, and *COL14A1* genes, and anterior cruciate ligament rupture [15,16,17,18,19].

The *COL1A1* gene is located on chromosome 17, at position 17q21.33. One of the most studied polymorphisms within this gene is the rs1800012, previously associated with ACL injuries [15,16,17]. This polymorphism results in the substitution of guanine for thymine, by increasing the affinity for the transcription factor Sp1, increasing the *COL1A1* gene expression, and leading to the synthesis of weaker collagen consisting of three type 1 α chains [20,21]. According to this hypothesis, individuals with the TT genotype should have a minor risk of ACL injuries [17].

The *COL5A1* gene is located on chromosome 9, at position 9q34.3. Mutations within this gene have been observed to lead to a reduction in connective tissue stiffness, and a 50% reduction in type V collagen fibers [22]. Among the most studied genetic variants within this gene are the rs12722 (BstUI) and the rs13946 (DpnII), which are both cytosine-to-thymine transitions. Previous research has demonstrated that the presence of cytosine (C allele) in both SNPs could represent a protective factor against ACL injury [23,24].

The *COL12A1* (6q13-q14.1) and *COL14A1* (8q24.12) genes encode type XII and type XIV collagens, respectively. These are a family of non-fibrillar collagens, referred to as fibril-associated collagens with interrupted triple helices (FACITs) [25].

Previous research studied the association of two polymorphisms within the *COL12A1* gene (the rs240736 and rs970547) with ACL rupture, and showed that women with the rs970547 AA genotype (corresponding to TT genotype in the 1000 Genome database) had an increased risk of ACL rupture [19].

However, a recent meta-analysis performed using five online databases (PubMed, EMBASE, ISI Web of Science, CENTRAL, and CNKI) with studies involving 1477 subjects with ACL rupture, and 100,439 healthy controls, concluded that the rs970547 polymorphism was not associated with ACL rupture risk in males, females, or the overall population among Asians or Caucasians [26].

Finally, the *COL14A1* gene interacts with the fibrillary surface, and participates in collagen biosynthesis, as well as in the degradation and regulation of fibrillogenesis [25], and some studies have shown an association between the *COL14A1* gene and ACL rupture [25,27].

The aim of the present study was to analyze the influence of six polymorphisms located in collagen genes (the *COL1A1* rs1800012; *COL5A1* BstUI rs12722; *COL5A1* DpnII rs13946; *COL12A1* rs240736; *COL12A1* rs970547; and *COL14A1* rs4870723) on ACL rupture in male and female athletes practicing team sports.

## 2. Materials and Methods

### 2.1. Sampling

A total of 181 athletes (*n* = 86 cases; *n* = 96 controls) were sampled for the study. There were 86 cases (*n* = 51 males, 28.4 ± 7.4 years; *n* = 35 females, 27.1 ± 6.4 years) with surgically-diagnosed ACL ruptures (cases), and 96 controls (*n* = 50 males, 26.7 ± 11.9 years; *n* = 46 females, 25.4 ± 8.9 years). The controls were all athletes from the same teams as the cases. All the injured athletes reported non-contact mechanisms of injury. All the participants were of European descent for at least three generations, and had played team sports (volleyball, football, and basketball) at a competitive level for at least ten years (ACL group, 11.6 ± 5.8 years; control group, 16.2 ± 7.9 years). Each participant completed a self-administered questionnaire, containing demographic and medical data, information about their sporting practice, and the mechanisms and dates of their ACL injury. The procedure followed in the study was conducted in accordance with the Declaration of Helsinki for Human Research of 1974 (last modified in 2000), and written informed consent was obtained from each participant. The Ethics Committee of the Azienda Ospedaliera Universitaria (AOU) of Cagliari University (Italy) approved the research.

### 2.2. Genotyping

A buccal swab was taken from each participant, and DNA was extracted using the salting-out method. The concentration and quality of the DNA were determined using the spectrophotometer NanoDrop (Thermo Scientific, Waltham, MA, USA). The extracted DNA was subsequently subjected to PCR. The SNPs of *COL5A1*, *COL12A1*, and *COL14A1* were genotyped through PCR RFLPs, as previously reported [28,29,30], whilst for the rs1800012 on the *COL1A1* gene, MTPA PCR was used [31]. The method was carried out using two internal primers (forward and reverse), and two external primers (forward and reverse), designed in silico using the specific software http://primer1.soton.ac.uk/primer1.html (accessed on 15 January 2020). The procedure was validated using the Sanger sequencing of 10 random samples.

### 2.3. Statistical Methods

The allele and genotype frequencies and group differentiation were tested through the Genepop program, ver. 4.4.3.

A comparison between the injured and non-injured groups was conducted using an unpaired Student’s *t*-test. SNPstats (https://snpstats.net/, accessed on 24 April 2023) was used to investigate the associations between each polymorphism and the ACL rupture, by means of a logistic regression analysis. The odds ratios (OR) and 95% confidence intervals (CI) were calculated under the co-dominant, dominant, and recessive models. Moreover, the AIC (Akaike information criteria) and BIC (Bayesian information criteria) were determined, to evaluate the most suitable genetic model.

Finally, the population branch statistic (PBS) was calculated, to detect possible signatures of natural selection in genes, using the R programming environment with specific R packages [32]. Firstly, only SNPs with MAF >= 0.05 were retained, using Plink 1.9 [33]; next, the FST of SNPs of paired populations was calculated, using VCF tools [34]; and finally, the PBS was calculated [35]. The Bonferroni correction was performed, to correct for multiple comparisons [36]. Thus, a *p*-value < 0.0083 (0.05/6) was considered statistically significant. For the Hardy–Weinberg (HW) equilibrium and baseline characteristics analysis, a *p*-value < 0.05 was regarded as statistically significant. Data are expressed as mean ± standard deviation (SD).

## 3. Results

All the genetic markers were in Hardy–Weinberg (HW) equilibrium among the cases and controls, with the only exception being the *COL5A1* BstUI (rs12722) in the control group. The lack of equilibrium of the rs12722 was due to a slight excess of observed homozygotes in the control group (*p* = 0.004). Therefore, the rs12722 was excluded from further statistical genetic analyses.

The allele frequency distribution in both the ACL and control groups fell within the world range (1000 Genomes dataset). When the Italian data were compared with European data, some peculiarities emerged.

In detail, the ACL group showed a decrease in the C allele frequency of the COL5A1 rs13946 polymorphism, and an increase in the A allele frequency of the COL14A1 rs4870723 polymorphism (*p* > 0.05). The COL12A1 rs970547 polymorphism showed a reduction in the T allele frequency, in both the ACL and control groups (Table 1).

The logistic regression analyses showed no association between the polymorphisms and ACL rupture (*p* > 0.008).

Knowing that the incidence of ACL rupture is greater in female than in male athletes [6,7], we performed a separate sex analysis. However, no association was found between ACL rupture in males and females, and each of the five polymorphisms under scrutiny (*p* > 0.008).

All the examined SNPs (with the only exception being the COL12A1 (rs970547)) showed a marked variability in the world distribution (data from 1000 Genomes).

**Figure 1:**
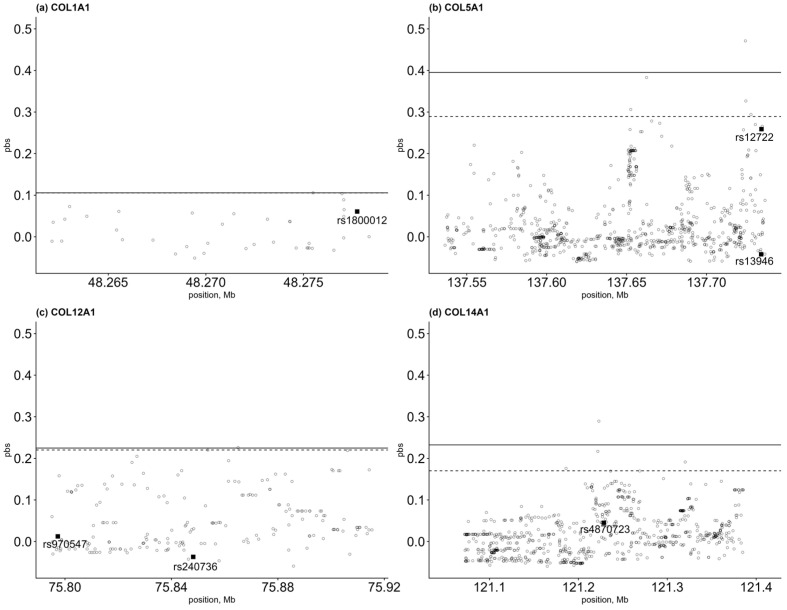
PBS representation of (**a**) *COL1A1*, (**b**) *COL5A1*, (**c**) *COL12A1*, and (**d**) *COL14A1*. The 99.5th (dotted horizontal lines) and 99.9th percentiles (continual horizontal lines) of the empirical distribution are shown in the plot. The SNPs under scrutiny are indicated with a square.

Through PBS, traces of selective pressure were detected for the genes under scrutiny. Although traces of selective events within the genes were found, in no case did the involved region include the selected SNPs, as can be observed in Figure 1a–d.

## 4. Discussion

While ACL rupture is one of the most common and serious musculoskeletal injuries, the exact mechanisms responsible for these acute injuries are still unknown. The majority of ACL ruptures occur in young athletes, particularly in sports requiring a change in direction and rapid deceleration during cutting, pivoting, and landing [37].

Research has demonstrated a familial predisposition to ACL rupture, which has been followed by genetic association studies on polymorphisms in candidate genes over the last few years, suggesting that genetic predisposition is an important factor in ACL rupture [38].

In the present study on athletes practicing team sports (volleyball, football, and basketball), the association of some polymorphisms within the *COL1A1*, *COL5A1*, *COL12A1*, and *COL14A1* genes with indirect ACL rupture was investigated. The main preliminary finding of our pilot study was that the distribution of allele and genotype frequencies between the cases and controls did not show any significant differences for any of the investigated variants (the rs1800012; rs13946; rs240736; rs970547; rs4870723) in genes encoding collagen.

Conversely, a different number of SNPs in collagen genes, such as *COL1A1*, *COL3A1*, *COL5A1*, and *COL12A1*, have been reported as candidate risk factors for ACL rupture, even if the evidence is contradictory and, actually, it is difficult to establish a clear link between genetic variants and the risk of ACL rupture [2,39].

A recent large-scale study analyzed the association of the genetic variants within the *COL5A1* gene with ligament injuries in physically active populations from three different countries, finding that the C/C genotype was under-represented in controls vs. the ACL rupture group in the Japanese population [23].

However, most of the genetic-association studies published to date are case-control studies, in which multiple sources of bias have been identified [39].

Alvarez-Romero et al. [23] found different results when populations from different origins were analyzed, underlining the importance of ethnicity in this type of case-control study.

A systematic review examined 33 different DNA variants associated with ACL rupture, and showed conflicting and limited evidence for an association between collagen genes and ACL rupture [2]. In particular, the authors found conflicting evidence for an association between ACL rupture, and the *COL1A1* rs1800012 and *COL3A1* rs1800255 polymorphisms. Moreover, limited evidence has been shown for there being any association between the *COL5A1* rs12722, *COL5A1* rs13946, and *COL12A1* rs970547 variants, and ACL rupture, while insufficient evidence has been demonstrated for any association between the *COL1A1* rs1107946, *COL6A1* rs35796750, and *COL12A1* rs240736 variants, and ACL rupture.

The preliminary results of the present study are not able to support previous findings on the association between genes coding for collagen proteins, and ACL rupture.

There could be many factors that may have influenced this. The first factor is represented by the small sample size used in the present study, which, due to the sex and genotypic stratification, reduced the chance of detecting a true effect. Moreover, our study was performed in Italian athlete populations, which may differ genetically from other populations. Last but not least, the differences in recruitment may have contributed to differences in the study populations, as the participating athletes in the present study all played team sports, and played competitively at a high level (national and international).

The main limitation of the present study is represented by the small sample size, as it is known that gene studies need more participants than most of those included in our study. Moreover, the sample size became very small when the groups were stratified by sex, by genotypes, and by condition (injured and non-injured). Finally, one more limitation is represented by the heterogeneity of the athletes in terms of the types of sport (volleyball, football, and basketball) included in the study, and the varying training exposure of the participants, which are both factors that could have influenced the results, and highlight the importance of a homogeneous sample.

Since most of the SNPs under scrutiny showed a marked variability in the world distribution, we verified that this variability could be the result of the action of natural selection, using PBS analysis. The traces of selective pressure detected never involved the selected SNPs; therefore, the variability found for all SNPs seems to be modeled more by the phenomena of genetic drift, rather than by the action of natural selection, in agreement with what previously attested for *COL14A1* [29].

## 5. Conclusions

In conclusion, the preliminary results of the present pilot study did not show any significant association between collagen genes and ACL rupture in Italian athletes, highlighting the need for further investigation in order to establish a clear association between ACL injury and genetic variants. Due to the low sample size, the results of this study should be interpreted with caution. Further genetic studies should involve a sufficient sample size, to achieve more confidence in the statistical power, and detect the true effect of SNPs on developing anterior cruciate ligament injuries induced by sports practice. In this way, genetic studies will be able to be used for diagnostic purposes, in order to create a personalized training program based on the genetic profile of the athletes. Major prospective studies are necessary to draw solid conclusions.

## Figures and Tables

**Table 1:** The allele and genotype frequencies in the ACL and control groups.

*COL1A1* rs1800012				
	All (*n* = 166)	ACL (*n* = 85)	Control (*n* = 81)	*p* value
G	0.720 (238)	0.685 (117)	0.750 (121)	0.281
T	0.280 (94)	0.315 (53)	0.250 (41)	
GG	0.490 (82)	0.450 (38)	0.540 (44)	0.250
GT	0.450 (74)	0.480 (41)	0.410 (33)	
TT	0.060 (10)	0.070 (6)	0.050 (4)	
*COL5A1* DpnII rs13946				
	All (*n* = 155)	ACL (*n* = 84)	Control (*n* = 71)	*p* value
C	0.790 (245)	0.180 (30)	0.250 (35)	0.165
T	0.210 (65)	0.820 (138)	0.750 (107)	
CC	0.080 (12)	0.060 (5)	0.100 (7)	0.203
CT	0.260 (41)	0.240 (20)	0.300 (21)	
TT	0.660 (102)	0.700 (59)	0.610 (43)	
*COL12A1* rs970547				
	All (*n* = 176)	ACL (*n* = 85)	Control (*n* = 91)	*p* value
T	0.540 (191)	0.550 (94)	0.530 (97)	0.746
C	0.460 (161)	0.450 (76)	0.470 (85)	
CC	0.200 (35)	0.180 (15)	0.220 (20)	0.744
TC	0.520 (91)	0.540 (46)	0.490 (45)	
TT	0.280 (50)	0.280 (24)	0.290 (26)	
*COL12A1* rs240736				
	All (*n* = 174)	ACL (*n* = 86)	Control (*n* = 85)	*p* value
A	0.790 (276)	0.770 (131)	0.810 (145)	0.354
G	0.210 (52)	0.230 (39)	0.190 (33)	
AA	0.600 (104)	0.550 (47)	0.640 (57)	0.299
AG	0.390 (68)	0.440 (37)	0.350 (31)	
GG	0.010 (2)	0.010 (1)	0.010 (1)	
*COL14A1* rs4870723				
	All (*n* = 164)	ACL (*n* = 84)	Control (*n* = 80)	*p* value
A	0.680 (223)	0.700 (117)	0.660 (106)	0.554
C	0.320 (105)	0.300 (51)	0.340 (54)	
AA	0.450 (73)	0.450 (38)	0.440 (35)	0.541
AC	0.470 (77)	0.490 (41)	0.450 (36)	
CC	0.090 (14)	0.060 (5)	0.110 (9)	

## Data Availability

The data used in this study are available in the Methods section of the manuscript. Individual data are available upon reasonable request from the corresponding author.
